# Micellar Aggregation Behavior of Alkylaryl Sulfonate Surfactants for Enhanced Oil Recovery

**DOI:** 10.3390/molecules24234325

**Published:** 2019-11-26

**Authors:** Huoxin Luan, Lingyan Gong, Xinjian Yue, Xiaobin Nie, Quansheng Chen, Dan Guan, Tingli Que, Guangzhi Liao, Xin Su, Yujun Feng

**Affiliations:** 1 Experimental Detection Research Institute, Xinjiang Oilfield Company, Karamay 834000, China; luanhuoxin@petrochina.com.cn (H.L.); nxb@petrochina.com.cn (X.N.); chenquansheng@petrochina.com.cn (Q.C.); guandan1@petrochina.com.cn (D.G.); quetingli1986@petrochina.com.cn (T.Q.); 2 Polymer Research Institute, State Key Laboratory of Polymer Materials Engineering, Sichuan University, Chengdu 610065, China; lingyangong22@163.com; 3 PetroChina Exploration & Production Company, Beijing 100007, China; liaoguangzhi@petrochina.com.cn

**Keywords:** enhanced oil recovery, alkylaryl sulfonate surfactant, critical micellar concentration, micellar aggregation behavior, surface tension

## Abstract

Alkylaryl sulfonate is a typical family of surfactants used for chemically enhanced oil recovery (EOR). While it has been widely used in surfactant–polymer flooding at Karamay Oilfield (40 °C, salinity 14,000 mg/L), its aggregation behavior in aqueous solutions and the contribution of aggregation to EOR have not been investigated so far. In this study, raw naphthenic arylsulfonate (NAS) and its purified derivatives, alkylaryl monosulfonate (AMS) and alkylaryl disulfonate (ADS), were examined under simulated temperature and salinity environment of Karamay reservoirs for their micellar aggregation behavior through measuring surface tension, micellar size, and micellar aggregation number. It was found that all three alkylaryl sulfonate surfactants could significantly lower the surface tension of their aqueous solutions. Also, it has been noted that an elevation both in temperature and salinity reduced the surface tension and critical micellar concentration. The results promote understanding of the performance of NAS and screening surfactants in EOR.

## 1. Introduction

Petroleum is an important strategic resource that plays a critical role in economic growth and improving living standards. However, extraction of crude oil becomes increasingly difficult owing to the entrap of residual oil [1,2,3]. A total of 60% of crude oil generally remains underground after self-flowing and secondary oil recovery by waterflooding [4,5]. Thus, tertiary oil recovery, also referred to as “enhanced oil recovery” (EOR), is used to produce more oil [6,7,8]. Among the various EOR techniques, chemical ones, particularly surfactant flooding, are vital as they can effectively decrease oil–water interfacial tension, thereby allowing oil droplets to flow more easily through pore throats because of reduced capillary trapping through increasing the capillary number (N_C_) [6].

N_C_ is defined as the ratio of viscous force to capillary force, and represents the relative effect of viscous drag force versus interfacial tension (IFT) acting across an interface between two immiscible liquids [6], as shown below:

(1) $$N_{c}=\frac{\nu \mu_{w}}{\gamma_{\mathrm{ow}}}$$

where μ_w_ and υ are the viscosity and interstitial velocity of displacing fluid, respectively; γ_ow_ is the IFT between crude oil and water.

The recovery of oil is dependent on the capillary and viscous forces present in the reservoir. After water flooding, N_C_ is generally in the range of 10^−7^−10^−6^, and if the residual oil saturation is decreased from 100% to about 70%, then N_C_ must be lowered from these values to 10^−4^. To achieve this target, the interfacial tension between oil and the displacing fluid must be decreased from about 30 to 10^−3^ mN/m or even lower. Thus, such an ultra-low IFT [7,8,9] is always pursued by formulating middle-phase microemulsions in the chemical EOR process while other factors, such as wettability alteration, are also believed to be the driving force for increasing oil recovery factor [10,11,12].

Wettability alteration results from a change in the hydrophilic/lipophilic properties of rock surface by the added surfactant [13,14,15]. According to Young’s equation [16], the wettability can be understood using the contact angle (θ) between the rock surface and an oil droplet covering this surface. If θ > 90°, then the surface of rock is hydrophobic, and if θ < 90°, it becomes hydrophilic. As reported by Banat [7], Hussein [8], and Harbottle [9], the added surfactant can adsorb on the substrate surface of the rock and change its hydrophilic/lipophilic properties. As the rock becomes more water-wet, wettability alteration occurs, which reduces the residual oil saturation. Then, water can flow from fractures into matrix blocks to displace oil, thereby changing the constraints to crude oil on the rock surface and improving the flow of crude oil. The application of surfactants to alter the wettability by decreasing the interfacial tension is one of the techniques adopted to recover oil from oil-wet reservoirs [16,17].

With the coexistence of oil and water phases, as the concentration of surfactant is increased to some extent, it is generally believed that Winsor Type I (lower phase), Type II (upper phase), or Type III (middle phase) microemulsions could be formed [3,4,5]. It is well recognized that the Type III microemulsion is necessary for decreasing IFT in the EOR process [5]. However, what we recently experienced seems to be an exceptional case. Naphthenic arylsulfonates (NAS) [18,19] derived from the local oil cracking components have been widely used in the polymer–surfactant process in Karamay Oilfield of PetroChina, and an 18% in situ recovery factor has been obtained [10], which is much higher than single polymer or surfactant flooding. The most important issue is that no middle-phase microemulsion was found in the various formulations with NAS solution (0.2 wt%) and local crude oil and brines. The fact that there was such a high oil recovery factor from field trial and with no microemulsion formulation being injected stimulates us to explore alternative mechanisms behind this observation. One of the aims to address this is to examine the role of the subcomponents of the raw NAS products, and we wondered if the 0.2 wt% NAS solution used in the displacing fluids could form swollen micelles to carry further oil during this process.

Therefore, in this work, we compared the basic properties of NAS and its alkylaryl monosulfonate (AMS) and alkylaryl disulfonate (ADS) derivates, in particular, their surface activity, micellar size, and micellar aggregation number, the latter two of which are strongly linked to the solubilization properties of micelles.

## 2. Results and Discussion

**Scheme 1:**
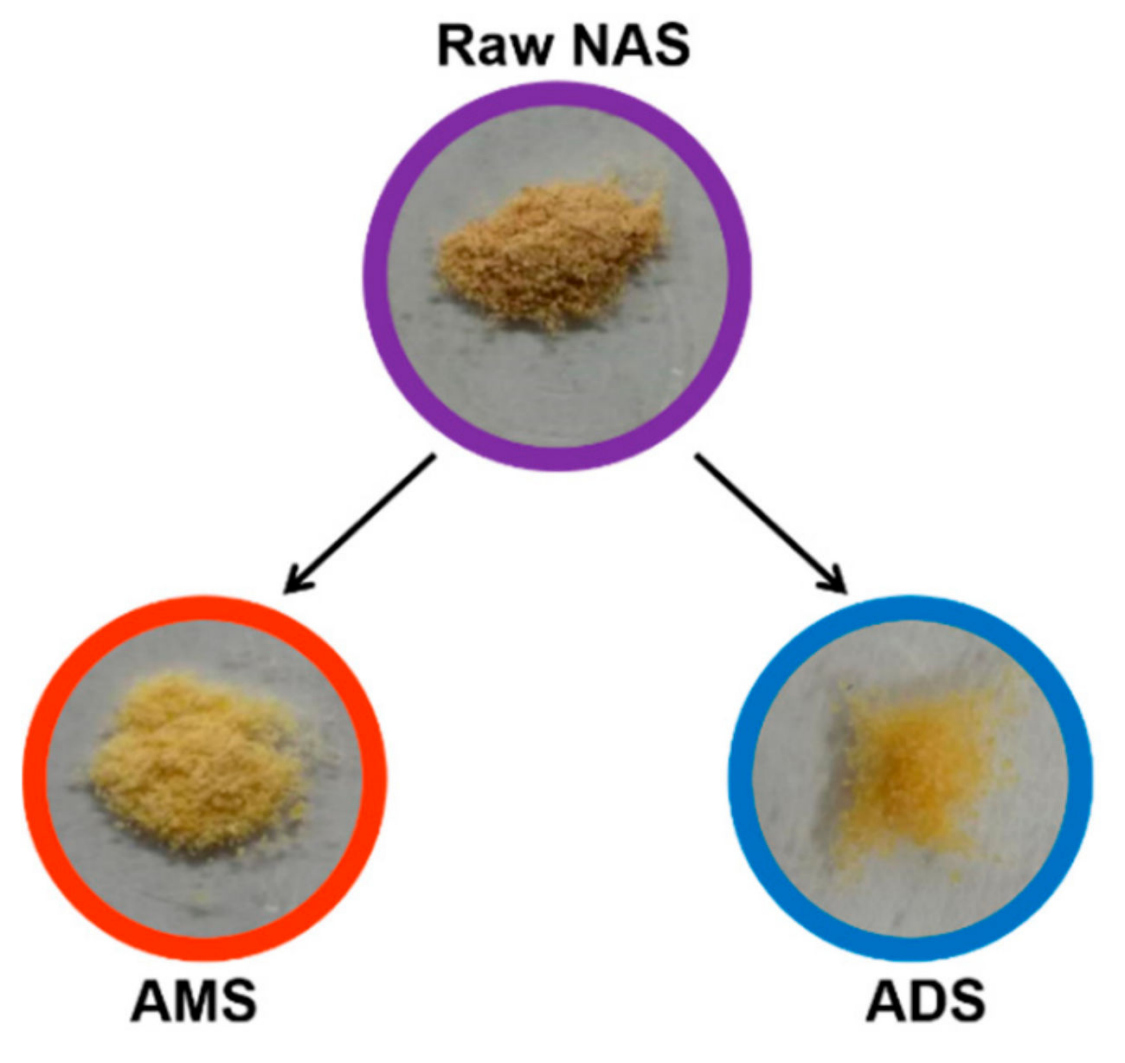
The appearance of raw naphthenic arylsulfonates (Raw NAS), alkylaryl monosulfonate (AMS), and alkylaryl disulfonate (ADS).

As shown in Scheme 1, the three surfactants, raw NAS, AMS, and ADS, appear as brown powders. Before studying their micellar behavior, their composition and molecular structures were analyzed and elucidated using GPC, ^1^H NMR, thermogravimetric analysis, and mass spectrometry, and the results are listed in Figures S1–S14 and summarized in Table 1. The raw NAS surfactants have relatively complex composition (Table 1), including benzenes, naphthalenes, alkyl indans, acenaphthenes, and alkane sulfonate. The main composition of ADS, which is separated from raw NAS, is sodium alkyl benzene sulfonate; AMS is the part that remains after separating ADS from raw NAS.

In order to simulate the oil reservoir environment of Karamay Oilfield [20], the test temperature was fixed at 40 °C, along with a salinity of 14,000 mg/L. Thus, this study investigated the concentration-dependent micellar aggregation behavior of three alkylaryl sulfonate surfactants under these temperature and salinity conditions.

### 2.1. Concentration Dependence of Micellar Aggregation Behavior

#### 2.1.1. Surface Tension

**Figure 1:**
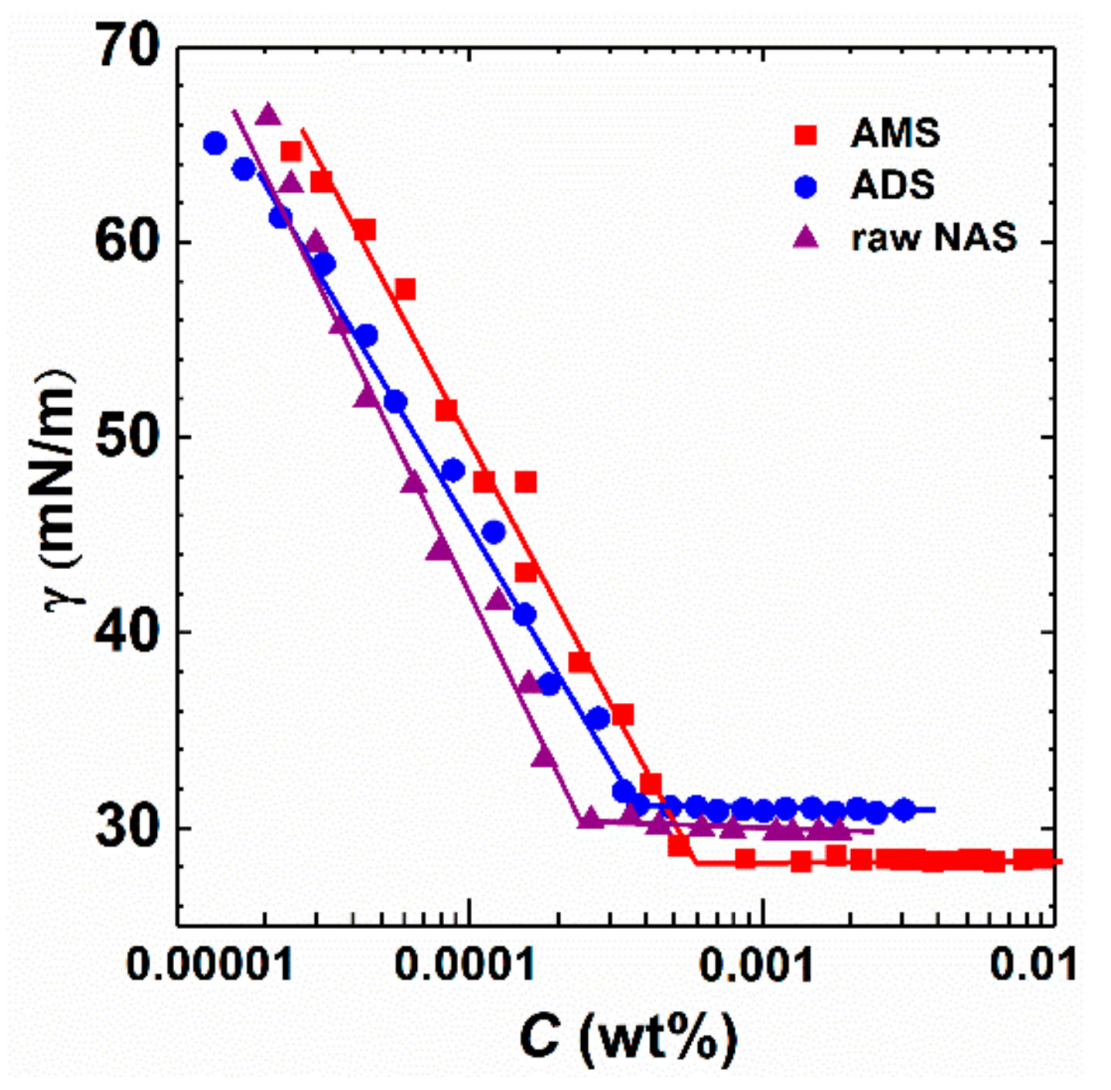
Surface tension as a function of concentration in the aqueous solutions of raw NAS, ADS, and AMS at 40 °C (salinity 14,000 mg/L).

**Figure 2:**
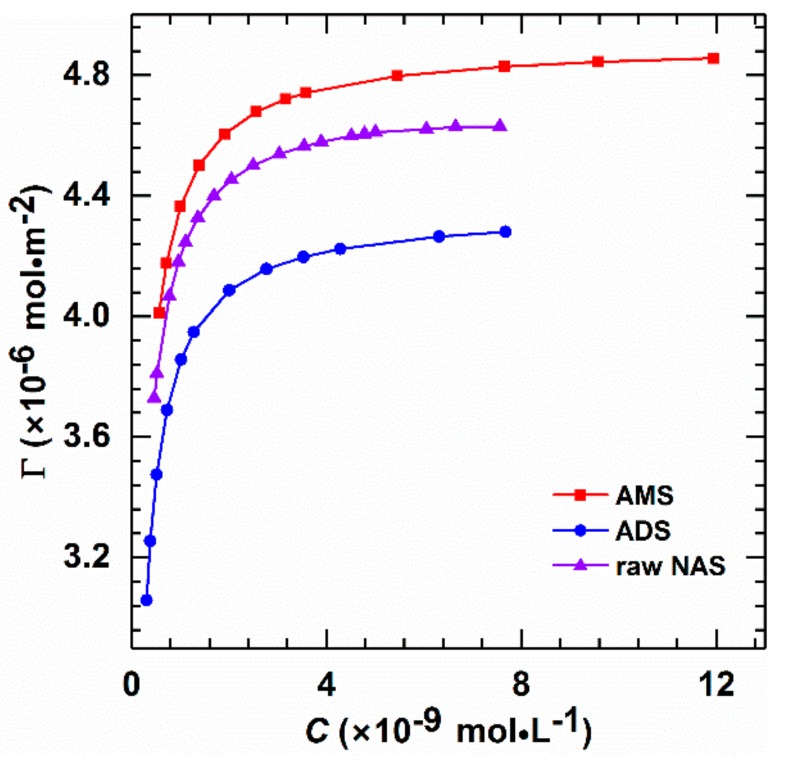
Langmuir adsorption isotherms of AMS, ADS, and raw NAS at 40 °C (salinity 14,000 mg/L). The molecular weights of all three surfactants are 400, as measured by GPC.

The surface tension of raw NAS, AMS, and ADS was measured at 40 °C (Figure 1). In aqueous solutions, the alkylaryl sulfonate molecules demonstrate surface adsorption and auto-agglutination. Under extremely low concentrations, the surface tension of these surfactant aqueous solutions is close to that of brine with salinity of 14,000 mg/L, indicating that only a few alkylaryl sulfonate molecules aggregate on the solution surface. A decrease in surface tension demonstrates aggregation of surfactant both on the bulk and in the solution. Surfactant molecules on the surface are gradually transformed from a loose arrangement to a dense state, resulting in a sharp reduction in the surface tension. When the concentration was increased further to the saturated adsorption, a tightly arranged monomolecular film formed at the air–water interface while the surfactant molecules in the solution coalesce and begin to form aggregates inside lipophilic groups and outside hydrophilic groups. Under these conditions, the surface tension reaches the minimum value. When the concentration of alkylaryl sulfonate reaches the critical micellar concentration (CMC), the surface tension of the solution remains the same. The CMCs of three alkylaryl sulfonates vary slightly, and the highest CMC is obtained from AMS followed by ADS and raw NAS. This is due to the length of hydrophobic chains in ADS, and those of raw NAS are longer than those of AMS (Table 1). Therefore, ADS and raw NAS easily form micelles and have lower CMCs than AMS. Raw NAS is a mixture of AMS and ADS, causing the lowest surface tension of raw NAS (30.4 mN/m), and is between those of AMS (29.2 mN/m) and ADS (30.7 mN/m). Figure 2 shows the Langmuir adsorption isotherms of three surfactants, and the maximum adsorption capacity at the air–water interface of raw NAS is between AMS and ADS. Though all the three surfactants are mixtures, their molecular weights are approximately 400 (Figures S7 and S8), which was measured using GPC and helpful for calculating the molar concentration for Langmuir adsorption isotherms (Figure 2).

#### 2.1.2. Micelle Aggregation Number

The aggregation behavior of surfactants in aqueous solution can be determined by the micelle aggregation number (N), which is derived from the fluorescence spectroscopy results with pyrene as the probe [21]. If the concentration of pyrene in solution is lower than 1 × 10^−2^ mmol/L, then the fluorescence spectrum has five peaks at wavelengths of 373, 373, 384, 390, and 397 nm [22]. The fluorescence intensity ratio of the first to that of the third peaks of pyrene (I_1_/I_3_) is generally referred to as hydrophobic factor [22,23]. The fluorescence intensity of pyrene is low because of its extremely low solubility. However, the solubility of pyrene increases significantly in a hydrophobic environment, which can enhance the fluorescence intensity. In this case, the pyrene molecules from water phase can enter the hydrophobic zone of micelles, which reveals the aggregation behavior of surfactant molecules in an aqueous solution.

**Figure 3:**
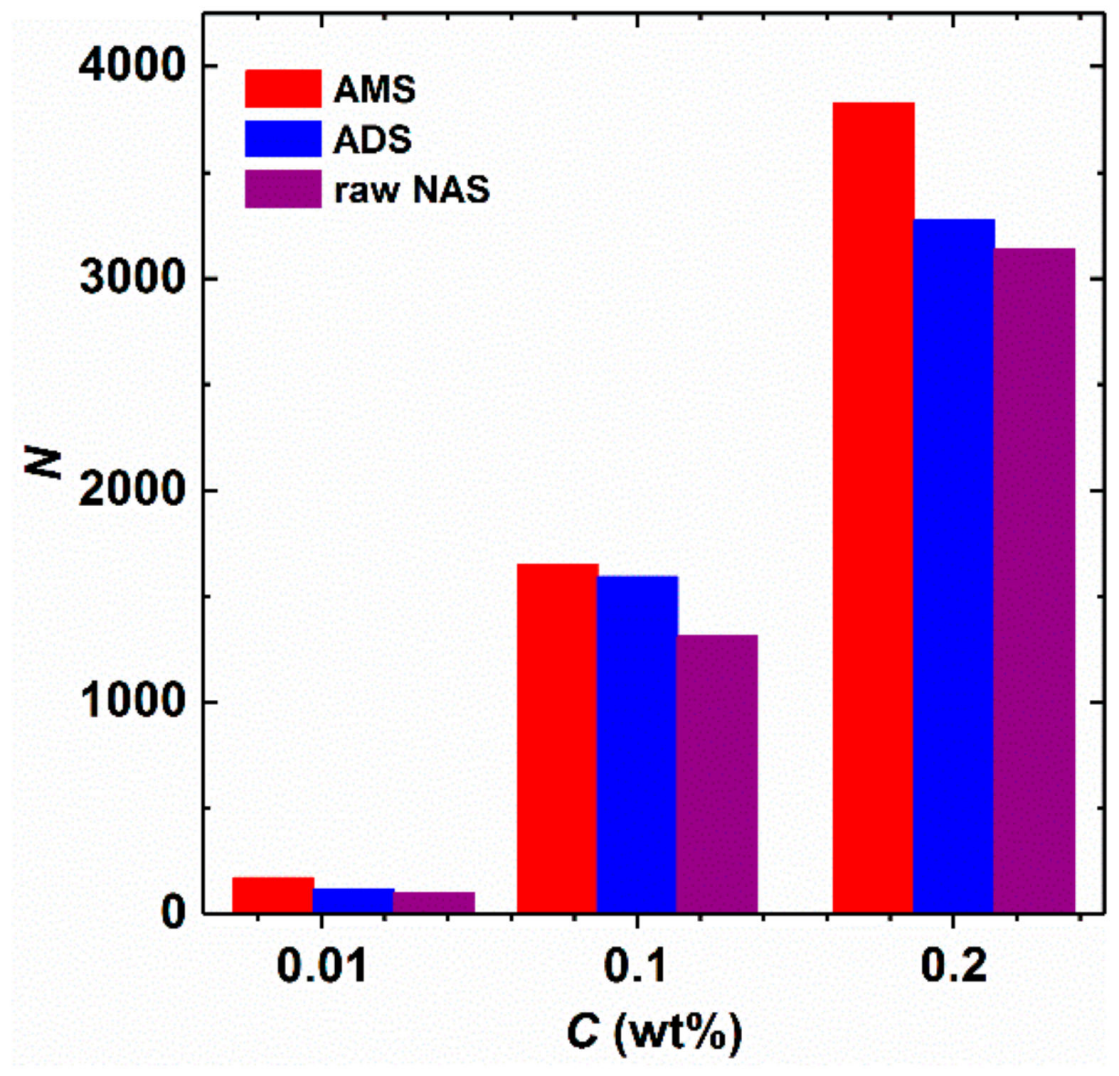
Micelle aggregation number as a function of concentration in the aqueous solutions of raw NAS, ADS, and AMS at 40 °C (salinity 14,000 mg/L).

The micelle aggregation number [24,25] of a spherical micelle is the average number of molecules aggregated in the micelle. The value of N was estimated from the decay fluorescence profile of pyrene (Figure S15). From Figure 3, the trend of variation in N with surfactant concentration can be found, and N is positively related to increased concentrations of surfactant. Given that alkylaryl sulfonate surfactants AMS and ADS have a high surface activity, their initial N is generally small at very low concentrations, but N increases significantly with an increase in surfactant concentration. As a result, the number of surfactant molecules in one micelle increases continuously as the surfactant concentration increases, thereby forming a micelle composed of dozens or hundreds of surfactant molecules. The hydrophobic group of surfactants is completely wrapped into the micelle, and the repulsive force between the hydrophobic group and water molecules diminishes and reaches the minimum. The high surface activity enhances the ability of single surfactant molecule to form micelles, causing N to increase. A higher N number indicates the presence of more hydrophilic groups on the surface of micelles, and more hydrophobic groups are hidden in the core of micelles [26,27,28].

#### 2.1.3. Micellar Size

**Figure 4:**
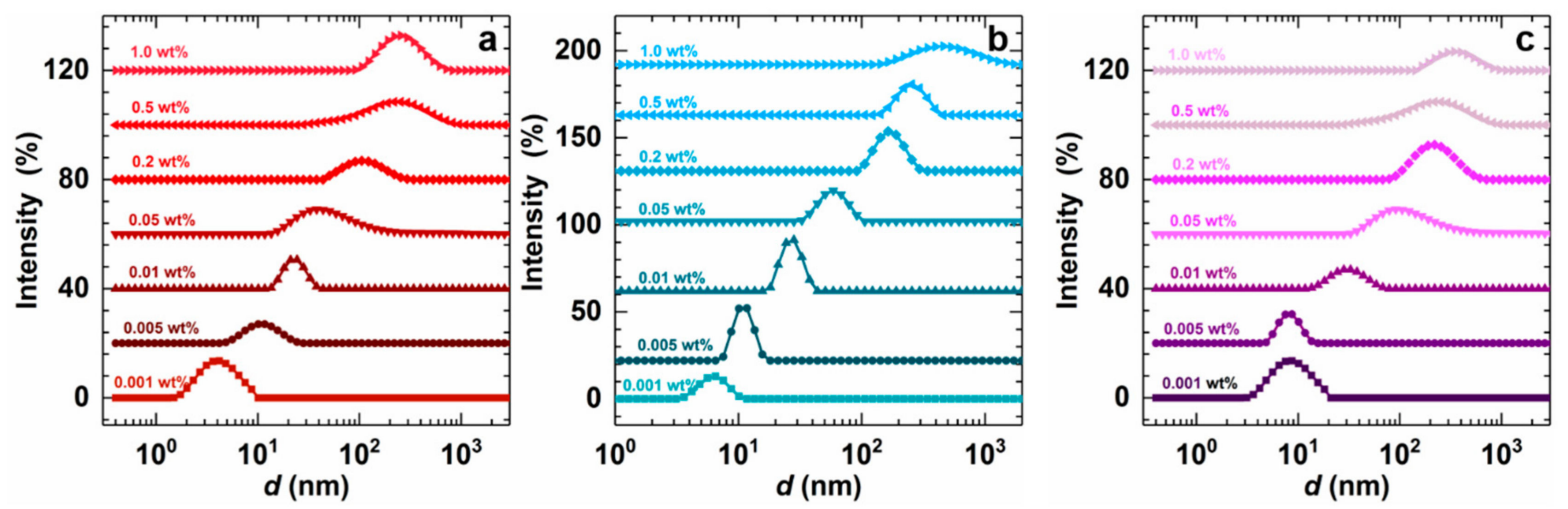
Effect of concentration of surfactant on the micellar size: (**a**) AMS, (**b**) ADS, and (**c**) raw NAS at 40 °C (salinity 14,000 mg/L).

**Figure 5:**
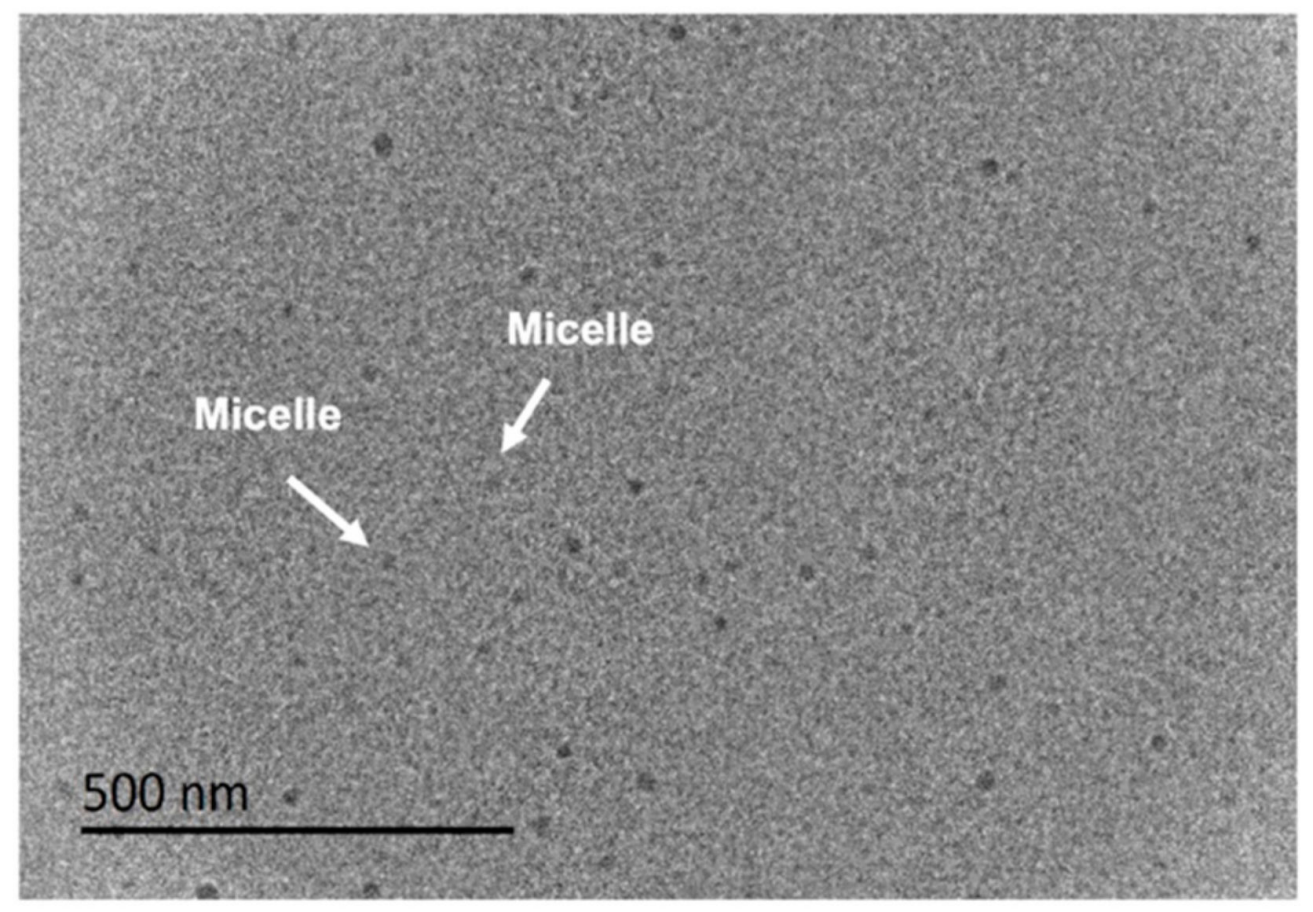
The Cryo-TEM image of 0.2 wt% aqueous AMS solution.

Figure 4 shows the distribution of micellar size under different surfactant concentrations. Micellar size increases gradually as the concentration of surfactant is increased. At low concentrations of surfactant, a few surfactant molecules aggregate to form micelles, the size of which is relatively small, and the agglomeration of surfactant molecules reaches approximately 100 nm as the concentration is continuously increased. An increase in the concentration of surfactant may also cause changes in morphology, thereby increasing the micellar size and N. The wider distribution peaks at different concentrations are also modified by changes in concentration, which is possibly due to changes in the morphology of aggregates. In Figure 4, it is shown that the micellar sizes of the three surfactants enhance significantly with increasing concentration. The micelles of AMS and ADS are smaller than raw NAS. At 0.2 wt%, the micelle sizes of AMS and ADS are 104 and 187 nm, respectively; but the micellar size of NAS at 0.2 wt% is 245 nm. The aqueous solution of 0.2 wt% AMS was observed by Cryo-TEM (Figure 5), and a sphere-like morphology was observed in the aggregates. The aqueous solution of AMS at 0.2 wt% was visualized by Cryo-TEM, and it was observed that the assembly of AMS in solution could be in the form of vesicles.

As summarized in Table 2, after separation, the three surfactants have similar properties with AMS demonstrating the best surface activity. AMS has the lowest surface tension when its concentration is 0.2 wt%, at which it has the largest aggregation number and the smallest micelle size. Therefore, AMS was chosen and used for further investigation.

### 2.2. Temperature Dependence of Micellar Aggregation Behavior

**Figure 6:**
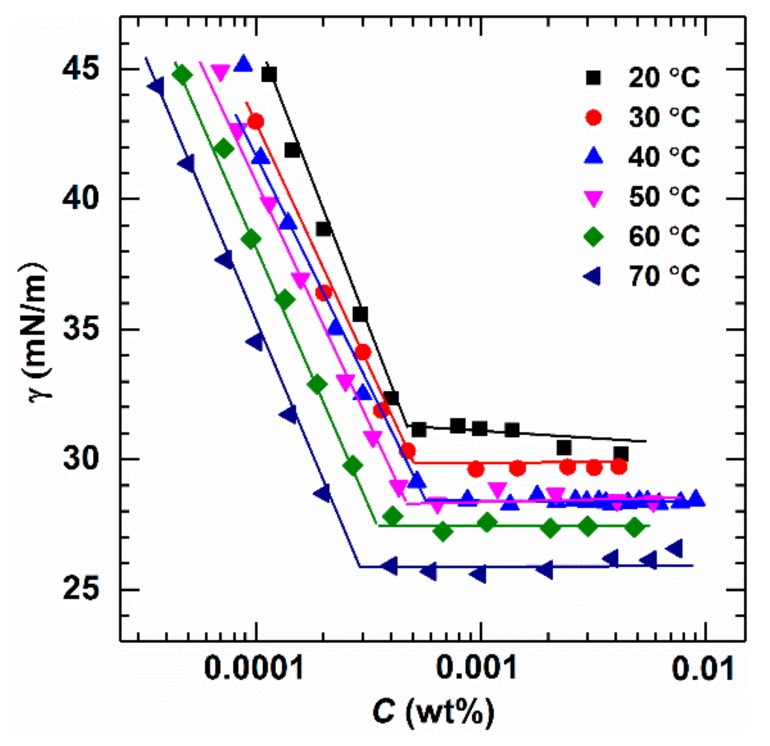
Surface tension as a function of concentration for aqueous AMS solutions under different temperatures (salinity 14,000 mg/L).

The aggregation behavior of micelles may be influenced by temperature variations during the displacing process. Thus, the influence of temperature on the micellar aggregation behavior of aqueous AMS solutions was examined in the range of 20–70 °C. The obtained results are depicted in Figure 6. This demonstrates a trend of consistent variations at different temperatures, showing higher temperature result in lower CMC and better surface activity.

Within a certain concentration range, the surface tension of surfactant solutions is reduced continuously as the concentration is increased. This is attributed to the hydrophilic groups of surfactant molecules interacting with water molecules and exhibiting arrangement in the water phase, and the hydrophobic groups of surfactants extending toward air due to the repulsive force from the water phase [29,30,31,32,33]. As a result, the surfactant molecules concentrate at the air–water interface and reduce the surface tension [32]. Surface tension does not change significantly when the concentration of surfactant reaches the CMC as the increased concentration of the solution could increase the number of micelles but not the number of adsorbed surfactant molecules on the interface after the formation of micelles [31,34]. It is observed that the surface tension decreases as temperature increases. Two factors govern surface tension: the interaction between liquid molecules, and the density difference between gas and liquid phases, and both of these factors are related to temperature. Hence, an increase in temperature weakens the intermolecular attraction, and the difference in density between the two phases is also reduced by the increase in temperature [35,36], resulting in lowering of the surface tension and CMC of aqueous AMS solutions.

From Figure 6, the slope ∂ln(CMC)/∂T at different temperatures can be calculated. The thermodynamic parameters such as the standard molar Gibbs free energy of micellization ($\Delta G_{m}^{\theta }$), standard molar enthalpy change of micellization ($\Delta H_{m}^{\theta }$), and standard molar entropy change of micellization ($\Delta S_{m}^{\theta }$) were calculated using Equations (2)–(4) [37]. The thermodynamic data of AMS are shown in Table 3.

(2) $$\Delta G_{m}^{\theta }=2\mathrm{RTln}\left(\mathrm{CMC}\right),$$

(3) $$\Delta H_{m}^{\theta }=-2{\mathrm{RT}}^{2}\left(\frac{\ln\left(\mathrm{CMC}\right)}{T}\right),$$

(4) $$\Delta S_{m}^{\theta }=\left(\Delta H_{m}^{\theta }-\Delta G_{m}^{\theta }\right)/T,$$

where CMC is expressed by the molar fraction, R is the molar gas constant, T is the absolute temperature, and $\ln\left(\mathrm{CMC}\right)$/T is the slope of ln(concentration)/temperature.

From Table 3, it can be observed that in the investigated temperature range $\Delta G_{m}^{\theta }$ is negative, indicating that the micellization of mono AMS in solution is spontaneous, and the formed micellar solution system is a thermodynamically stable system [38,39]. Based on Equation (2), an increase in temperature should reduce the value of CMC, which matches the trend as indicated in Figure 6. $\Delta H_{m}^{\theta }$ is negative, revealing that the entire micellization process is an exothermic process [38,40]. $\Delta S_{m}^{\theta }$ is positive, suggesting that the formation of micelles by the surfactant molecules is a spontaneous process [30] which is mainly driven by entropy and the collapse of the “iceberg structure” surrounding the alkane chain of surfactant molecules, making water molecules tend toward a disordered state [41].

**Figure 7:**
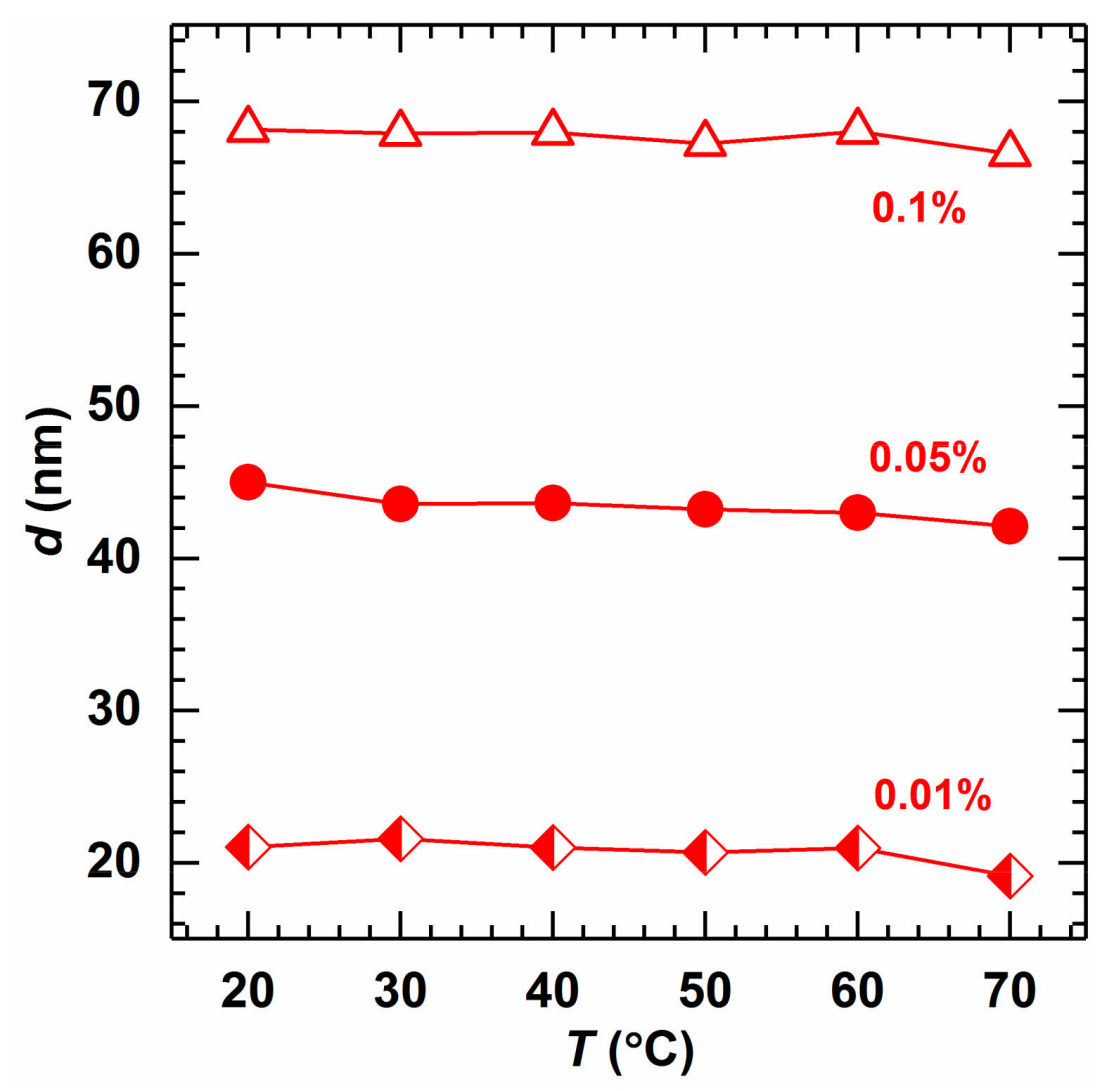
Effect of temperature on the micellar size at different concentrations of AMS (salinity 14,000 mg/L).

Figure 7 shows the influence of temperature on the micellar size of AMS. It can be found that the micelle size does not change with an increase in temperature. Instead, the change in temperature merely accelerates the movement of surfactant molecules, making it easier to form micelles without affecting the size of the micelles [42,43].

### 2.3. Salinity Dependence of Micellar Aggregation Behavior

Inorganic salts are always encountered in the EOR process, mainly from the dissolved rock in the connate water in reservoir formation underground. Moreover, the salinity is generally varied from the surface to the target oil-containing layer, so it is also necessary to check the impact of salt concentration on surface activity and aggregation behavior. As the major electrolyte found in Karamay Oilfield is NaCl, in this section, we thus mainly investigated the effect of NaCl content.

**Figure 8:**
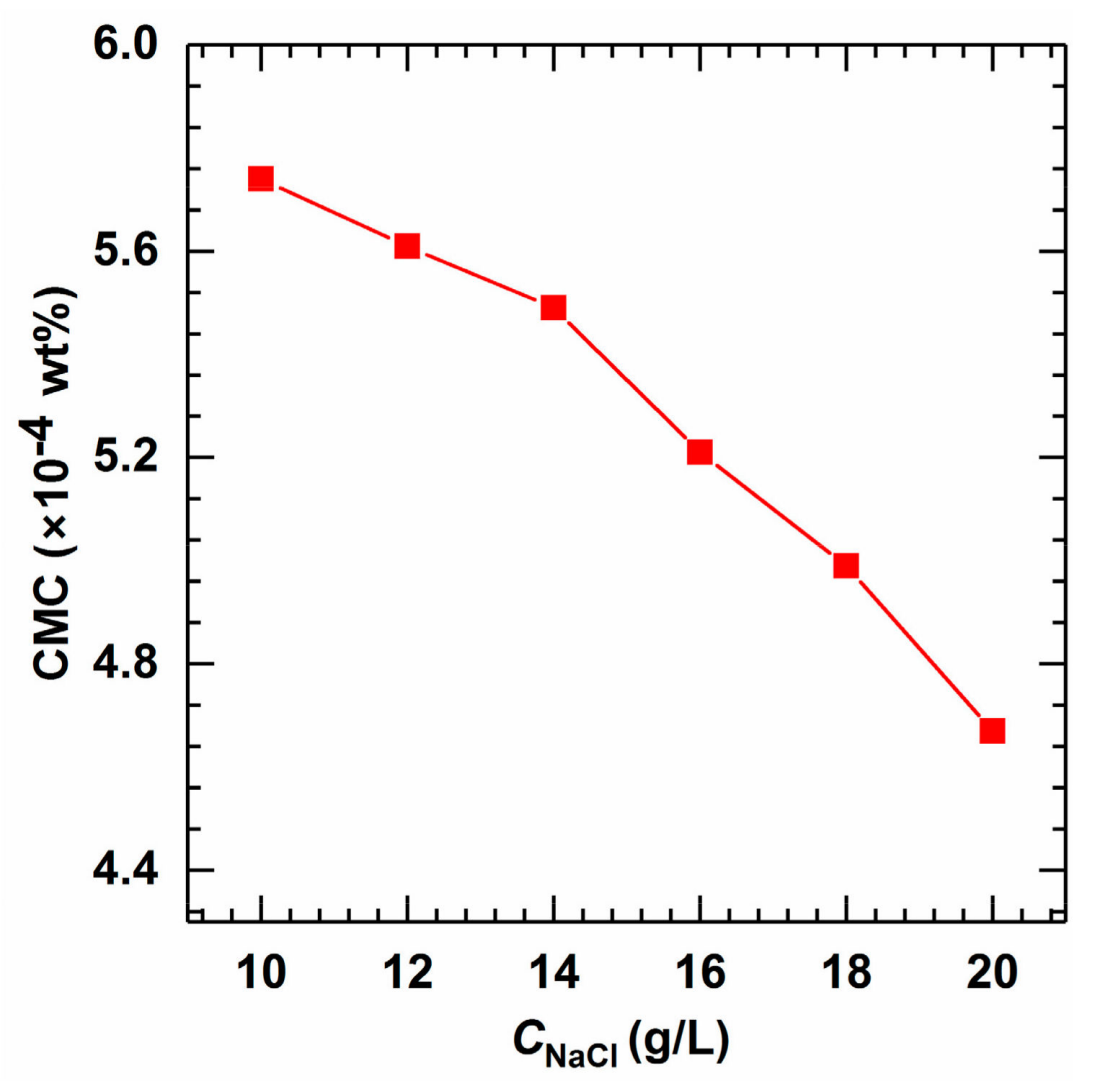
CMC as a function of salinity in the aqueous solutions of AMS at 40 °C.

It can be seen from Figure 8 that CMC decreases with the increase in NaCl concentration as the added salt might weaken the repulsive force between the headgroups and compress the diffuse double layer structure of the micelles, which is good for the formation of micelles [44,45]. Also, the addition of salt leads to diffusion-limited adsorption, demonstrating that the equilibration inside the solution is much slower than at the interface.

**Figure 9:**
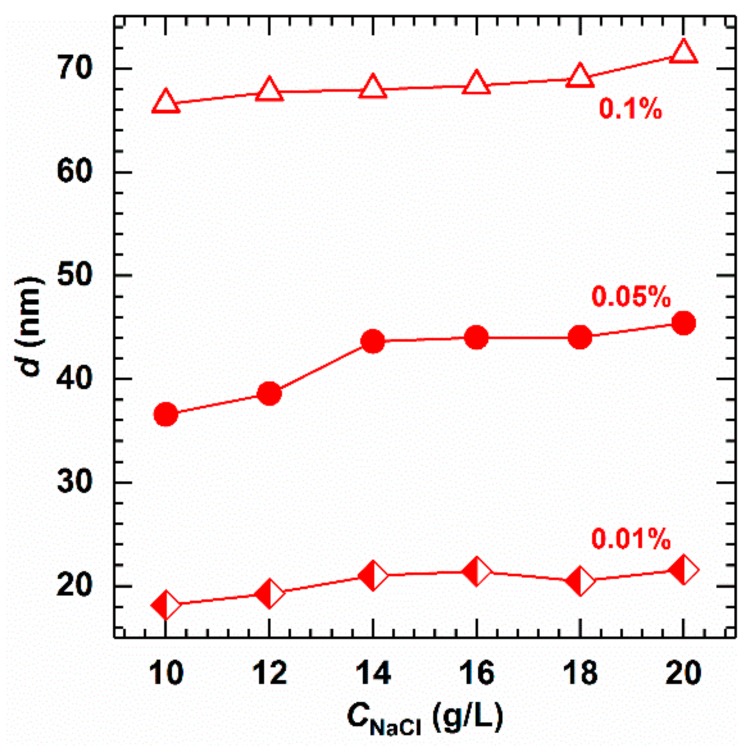
Effect of salinity on the micellar size at different concentrations of AMS at 40 °C.

Similarly, the effect of salinity on the micellar size of AMS was examined. As can be seen from Figure 9, the micellar size increases with an increase in added salt. This is due to the the reduced repulsion between the ion heads of surfactant by inorganic salt. Furthermore, the added inorganic salt assists surfactant with aggregating into larger micelles more easily.

### 2.4. Core Flooding Test

**Figure 10:**
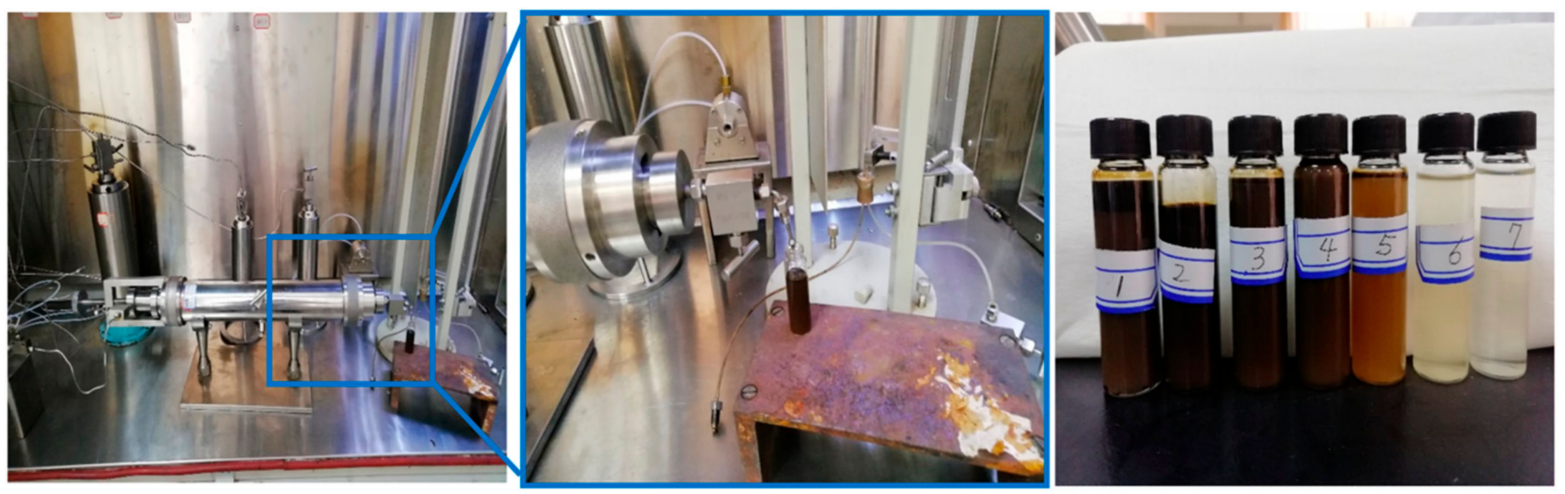
Core flooding experimental apparatus (left); The fluids produced at different times during the surfactant flooding process were collected (center); Starting from the first drop of oil at the outlet, samples #1–#7 were collected at regular intervals of 10 min (right).

To verify the change in the micellar size during injection of polymer–surfactant binary system, the core flooding test was performed (Figure 10). AMS was utilized in the test as it has the lowest surface tension and the largest aggregation number. The produced fluid was collected at different intervals during the core flooding process, and the particle size was measured by DSL (Figure S16). As the amount of solubilized crude oil gradually increases, the micelles of AMS swell, becoming as large as 1000 nm, and emulsion droplets finally form as a larger aggregate. Samples #6 and #7 demonstrated that after 50 min, no crude oil was collected, and the micellar size returned to the original size of AMS of about 100 nm.

## 3. Materials and Methods

### 3.1. Materials

The raw NAS and purified AMS, ADS were kindly offered by Prof. Yong Guo from Lanzhou Institute of Chemical Physics, Chinese Academy of Sciences (Lanzhou, China). Pyrene was purchased from Sigma-Aldrich Company (St. Louis, Missouri, USA). NaCl was supplied from Chengdu Kelong Chemicals Co., Ltd. (Chengdu, China). Ultrapure water (18.25 MΩ·cm) was prepared using a UPH-I-10T ultrapure water system (Chengdu Science and Technology Co., Ltd., China). Partially hydrolyzed polyacrylamide (HPAM, molecular weight of 10,000 Da), crude oil, and cylindrical artificial core were supplied by Xinjiang Oilfield Company (Karamay, China).

### 3.2. Characterizations

Surface tensiometry: For measuring the critical micelle concentration (CMC), Krüss K100 full-automatic surface tensiometer (Hamburg, Germany) was calibrated with triply -distilled water (surface tension of 69.56 ± 0.1 mN/m at 40 °C) before being subjected to any measurements. The concentration of the mother liquor was set to 10–20 times higher than the CMC. A program was set to add the mother liquor to saline water to gradually increase surfactant concentration. Variations in the surface tension with time under different concentrations were recorded. When the standard deviation of five successive measuring points was smaller than 0.05 mN/m, it was defined as the equilibrium surface tension of the solution.

Dynamic light scattering: The hydrodynamic size of micelles was measured using Malvern Zetasizer Nano ZS (Malvern, UK). To avoid the influence of the extreme large particles or insoluble components in the surfactant products, the as-prepared surfactant solutions were filtrated using a 0.8 μm filter. The experimental temperature was set at 40 °C, and each sample was tested at least three times to obtain an average value.

Fluorescence spectroscopy: The micelle aggregation number was estimated from the decay profile of pyrene fluorescence [46,47]. The probe pyrene was used because its monomer can form an association complex or excimer to determine the microenvironment of micelles. If the excimer is present, the fluorescence decay curve shows two components: a fast one associated with micelles having solubilized two or more pyrene molecules, and a slow one corresponding to the decay of the pyrene monomer.

Quenching is assumed to follow a first-order rate process, and the time dependence of fluorescence intensity is given as follows:

$$\ln\left[I_{\left(t\right)}/I_{\left(0\right)}\right]=-k_{1}t-n\left\{1-\exp\left(-k_{q}t\right)\right\},$$

where I_(0)_ is the fluorescence intensity at zero time, k_1_ is the first-order rate constant for unquenched fluorescence, n is the average number of quenchers per micelle, and k_q_ is the first-order quenching rate constant. It should be noted that Equation (5) is valid for the immobile reactants, i.e., when the residence time of the fluorescent molecule and the quencher inside the micelle is longer than the unquenched lifetime of the fluorescent probe.

For a very long time, when all the excimer decays, it is simplified to equation (6) below:

(6) $$\ln\left[I_{\left(t\right)}/I_{\left(0\right)}\right]=-k_{1}t-n.$$

The limiting slope of a plot of ln[I_(t)_/I_(0)_] vs. t is −k_1_. The extrapolation of the linear region to t = 0 is −n. A series of curves with varying concentration of quencher (excimer) produce parallel and linear tails in the logarithmic representation. This shows that no migration of the quencher occurs, as inherent in Equation (7), and from the value of n, the micelle aggregation number, N, is calculated:

(7) $$N=n\left(C_{s}-\mathrm{CMC}\right)/C_{\mathrm{py}},$$

where C_s_ is the total surfactant concentration and C_py_ is the concentration of pyrene. An effective micelle aggregation number can be determined and characterized by k_1_, can be identified. Decays were shifted to t = 0, which was considered to be the maximum for the fluorescence intensity, I_(0)_. A linear regression analysis provides the slope, −k_1_, and the intercept, −n. 

Cryo-TEM: The specimen was transferred to a Tecnai G2 F20 cryo-microscope (Hillsboro, Oregon, USA) using Gatan 626 cryo-holder and its workstation. The acceleration voltage was 200 kV, and the working temperature was kept below −170 °C.

### 3.3. Core Flooding Test

The oil displacement test was performed on a DQ-IV core flooding apparatus (Jiangsu Huaan Scientific Instrument Co., Ltd., Haian, China). The pore volume (PV) of the cylindrical artificial core was 31.4 mL, and the permeability (*K*_w_) was 123 mD. When the flooding system was maintained at 40 °C, the saturated core was injected with crude oil to achieve the oil saturation. Then, three-stage tests were performed: brine (salinity 14,000 mg/L) was first injected to get water cut of 98%; then a 0.5 PV (half of the core pore volume) 0.15% HPAM solution was injected; thirdly, the 0.2 wt% AMS solution was performed until no more oil was produced. The solvent for both polymer and surfactant was saline water with 14,000 mg/L NaCl. In the experiments, the injection rate of the displacing slug was controlled at 0.5 mL/min. For analyzing the interaction of polymer and surfactant, the collected samples were measured using Malvern Zetasizer Nano ZS (Malvern, UK) without removing the impurities.

## 4. Conclusions

The raw NAS surfactant product used in polymer–surfactant flooding in Karamay Oilfield was purified and separated into two different types of surfactants, i.e., AMS and ADS. To compare their micellar behavior in the aqueous solutions, these three alkylaryl sulfonate surfactants were studied. This revealed that the surface tension was approximately 30 mN/m at the CMC of the three surfactants in their aqueous solution with salinity of 14,000 mg/L. This confirms that all three surfactants have good surface activity and can be adsorbed at the air–water surface or spontaneously micellize in water. The number of micellar aggregates was positively related to the concentration of surfactants. The initial number of micellar aggregates was generally small as the alkylaryl sulfonate surfactants possess a high surface activity. As the concentration of surfactant increases, the micelle aggregation number increased significantly. The higher concentration of surfactant may cause morphological changes, and increase the micellar size as well as the micelle aggregation number. Surface tension and CMC are negatively correlated with temperature and salinity. During the core flooding test, the micelle of AMS was swollen as large as 1000 nm with crude oil; but when no crude oil was collected, and the micellar size remained at about 100 nm. After the separation, AMS had the lowest surface tension and the largest aggregation number. The obtained results provide a good basis for understanding the mechanism for the usage of NAS in EOR, and establishes a foundation for further studies. In the next stage, studies will be carried out to examine whether the recovery factor can be increased by solubilizing oil or its components with surfactant micelles.

## Figures and Tables

**Table 1:** The composition and molecular structures of three surfactants

Surfactants	Main Composition	General Molecular Structure
Alkylaryl monosulfonate (AMS)	Dominated by benzodicyclohexane sulfonate, supplemented by phenanthrene sulfonate, acenaphthene sulfonate, alkylbenzene sulfonate, indan sulfonate, and alkyl naphthalene sulfonate	Alkyl benzene sulfonate: 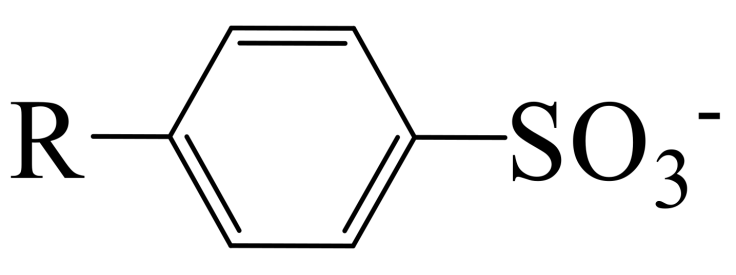 Alkyl indan sulfonate: 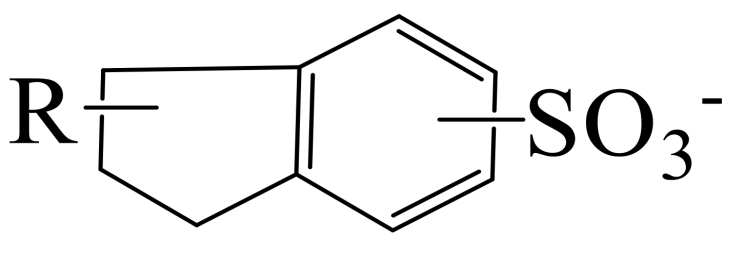 Alkyl naphthalene sulfonate: 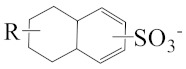 Alkyl naphthalene sulfonate: 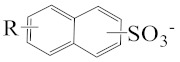 Benzodicyclohexane sulfonate: 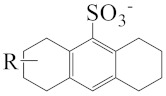 Alkyl acenaphthene sulfonate: 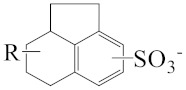
Alkylaryl disulfonate (ADS)	C_15-22_ sodium alkyl benzene sulfonate	
Raw naphthenic arylsulfonates (Raw NAS)	Dominated by benzodicyclohexane sulfonate, supplemented by phenanthrene sulfonate, acenaphthene sulfonate, alkylbenzene sulfonate, indan sulfonate, and alkyl naphthalene sulfonate	

**Table 2:** Comparison of AMS, ADS, and raw NAS.

Surfactant	CMC (× 10^−4^ wt%)	γ (mN/m) ^a^	N (C_surfactant_ = 0.2 wt%) ^b^	d (nm, C_surfactant_ = 0.2 wt%) ^c^
AMS	6.0	29.2	3890	104
ADS	3.7	30.7	3273	167
Raw NAS	2.4	30.4	3170	224

^a^ surface tension at critical micellar concentration (CMC); ^b^ aggregation number at 0.2 wt%; ^c^ micellar size at 0.2 wt%.

**Table 3:** Thermodynamic parameters of AMS in an aqueous solution during micellization (salinity 14,000 mg/L).

T/K	$\Delta G_{m}^{\theta }$	$\Delta H_{m}^{\theta }$	$\Delta S_{m}^{\theta }$	$-T\Delta S_{m}^{\theta }$
	(kJ·mol^−1^)	(kJ·mol^−1^)	(kJ·mol^−1^·K^−1^)	(kJ·mol^−1^)
293	−55.87	−5.26	0.1654	−49.24
303	−56.98	−14.13	0.1721	−44.22
313	−58.73	−15.49	0.1436	−43.26
323	−60.51	−6.71	0.1552	−53.48
333	−61.86	−9.31	0.1578	−54.75
343	−63.24	−11.94	0.1463	−52.29

## Supplementary Materials

The supplementary information is available online.
