# Supplementary material for: Micellar Aggregation Behavior of Alkylaryl Sulfonate Surfactants for Enhanced Oil Recovery
Source: Molecules. 2019 Nov 26;24(23):4325. doi: 10.3390/molecules24234325 (PMC6930474; doi:10.3390/molecules24234325)
Supplement: Supplementary file 1 [file molecules-24-04325-s001.pdf]

molecules

Supporting Information

Micellar Aggregation Behavior of Alkylaryl Sulfonate Surfactants for Enhanced Oil Recovery

Huoxin Luan [1], Lingyan Gong [2], Xinjian Yue [1], Xiaobin Nie [1], Quansheng Chen [1], Dan Guan [1], Tingli Que [1], Guangzhi Liao [3], Xin Su [2,*] and Yujun Feng [2,*]

[1] Experimental Detection Research Institute, Xinjiang Oilfield Company, Karamay, Xinjiang 834000, China
[2] Polymer Research Institute, State Key Laboratory of Polymer Materials Engineering, Sichuan University, Chengdu 610065, China
[3] PetroChina Exploration & Production Company, Beijing 100007, China
* Correspondence:xinsu@scu.edu.cn (X.S.); yjfeng@scu.edu.cn (Y.F.)

Content:

1. Infrared spectra....................2

2. ^{1}H NMR spectra....................3

3. GPC Results....................4

4. Thermogravimetric analysis (TGA)....................5

5. Mass spectrometry (MS)....................6

6. Aggregation number....................8

7. Micellar or particle size....................8

1. Infrared spectra

Infrared spectra of raw NAS, AMS, and ADS samples were recorded with Abb Inc-MB 3000 FTIR spectrophotometer. Figures S1S3 show that the peaks at 3441–3445 cm^{-1} are from the stretching vibrations of water molecules. The peaks around 2925 cm^{-1} are the stretching vibration adsorption of C–H bonds. The peaks at 1583–1630 and 1442–1458 cm^{-1} display the stretching vibration of the skeleton of the benzene ring. The peaks at 1182–1186, 1050–1058, and 632–644 cm^{-1} were the standard peaks for sulfonate groups. The infrared analysis indicates that the sodium petroleum sulfonate is the main component of the surfactant.

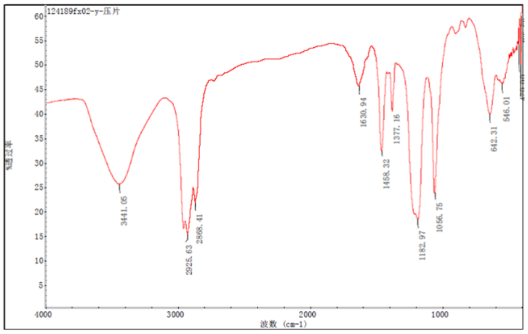

Figure S1. Infrared spectrum of AMS.

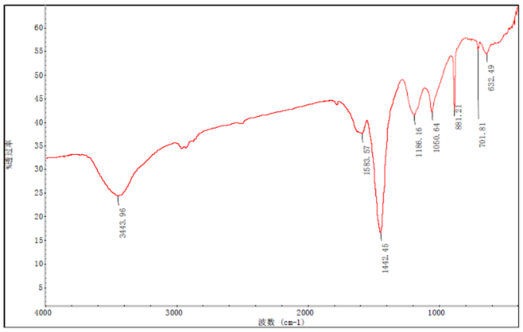

Figure S2. Infrared spectrum of ADS.

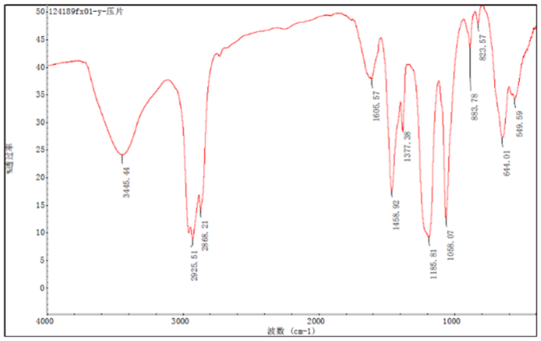

Figure S3. Infrared spectrum of raw NAS.

2. ¹H NMR spectra

The ¹H NMR spectra were obtained on a Bruker Avance-II 400 MHz NMR spectrometer. The ¹H NMR spectra of raw NAS, AMS, and ADS samples are shown in Figures S4–S6.

The ¹H NMR spectrum of AMS is shown in Figure S4, where the peaks at 0.87, 1.26, 2.38, 3.62, 6.74, and 7.58 ppm are the chemical shifts of the sodium petroleum sulfonate. The peaks at 3.57, 1.50, 1.31, and 7.58 ppm are the chemical shifts of n-butyl alcohol.

The ¹H NMR spectrum of ADS is demonstrated in Figure S5, where 0.80, 1.20, 2.50, 3.60, and 7.00 ppm are the chemical shifts of sodium petroleum sulfonate.

The ¹H NMR spectrum of raw NAS is displayed in Figure S6, where the peaks at 0.87, 1.26, 2.38, 3.62, 6.74, and 7.58 ppm are the chemical shifts of sodium petroleum sulfonate.

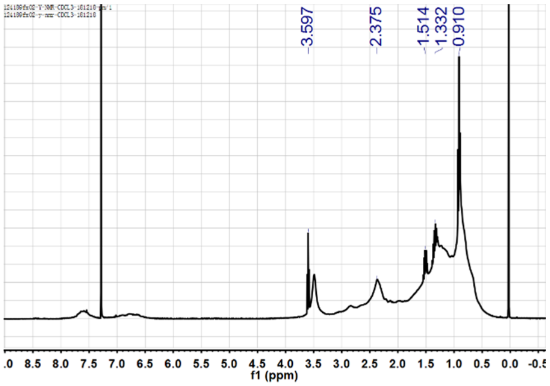

Figure S4. ¹H NMR of AMS.

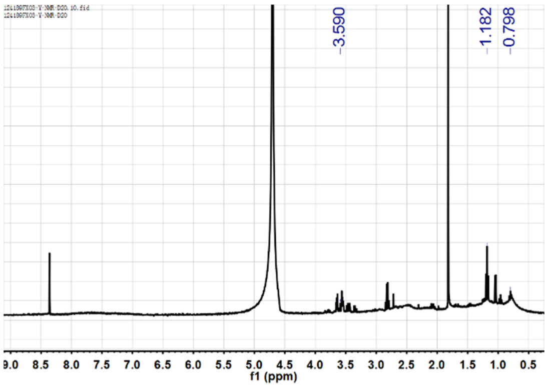

Figure S5. ¹H NMR of ADS.

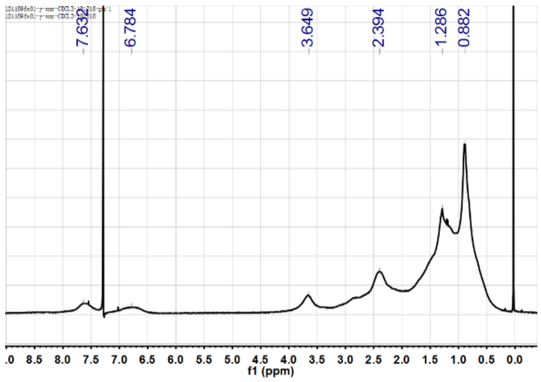

Figure S6. ¹H NMR of raw NAS.

3. GPC Results

Molecular weight data are essential for studying the chemical composition of surfactant for enhanced oil recovery (EOR). Given that the studied surfactants are three types of different complicated mixtures, the molecular weights of the contained compounds are not the same, and there is a wide range in molecular weights. Hence, only the average molecular weight could be used to represent the surfactant. GPC measurements (Shodex 3000 GPC system consisting of an HPLC pump) were performed to measure the molecular weight of samples. According to GPC, the molecular weight of sodium petroleum sulfonate in AMS and ADS was mainly distributed at approximately 400 D (Figures S7–S8).

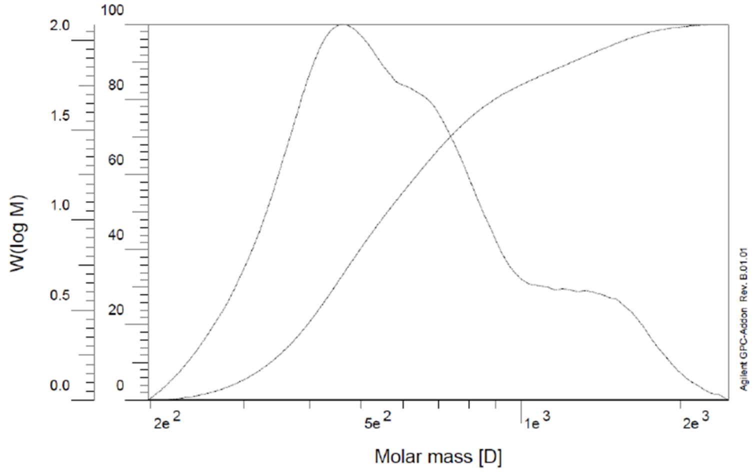

Figure S7. GPC test results of AMS.

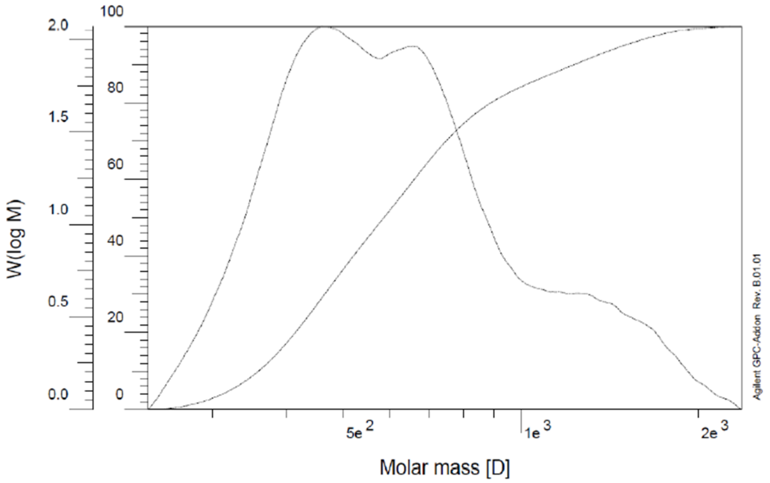

Figure S8. GPC test results of ADS.

4. Thermogravimetric Analysis (TGA)

According on the analysis above, the three surfactant samples all contain sodium petroleum sulfonate. The TGA on the samples was carried out to determine the thermostability of sodium petroleum sulfonate. The measurement was performed by TGA 2850 thermogravimetric analyzer (TA instruments) under N_2 in the temperature range 0–800 °C with an increase rate of 5 °C/min. The TGA results of the three types of dried surfactant samples are shown in Figures S9–S11.

For AMS, the dialysis temperatures of n-butyl alcohol and sodium petroleum sulfonate tested by TGA are 138 and 456 °C, respectively.

For ADS, the dialysis temperatures of sodium petroleum sulfonate and sodium carbonate tested by TGA are 444 and 717 °C.

For raw NAS, the dialysis temperature of sodium petroleum sulfonate tested by TGA is 447 °C.

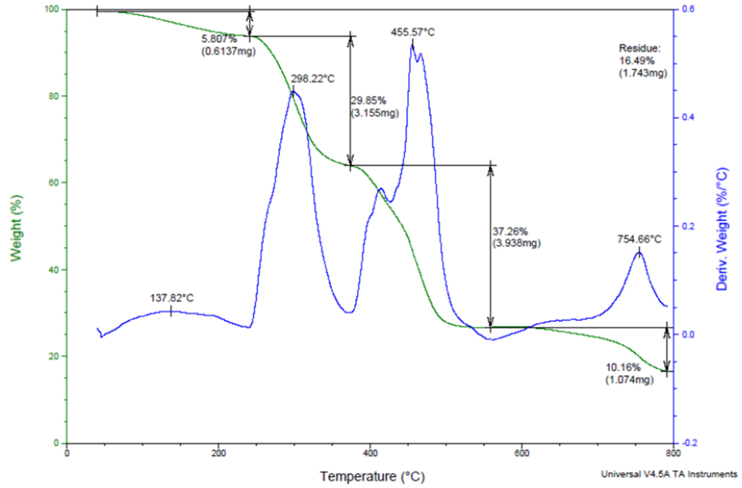

Figure S9. TGA results of dried AMS.

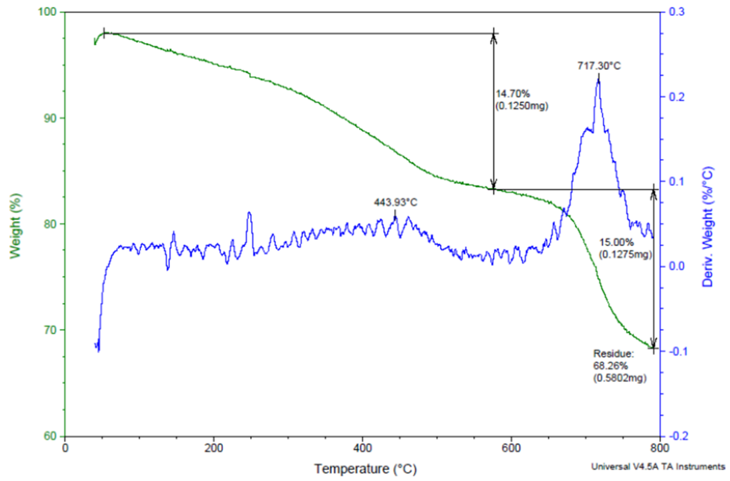

Figure S10. TGA results of dried ADS.

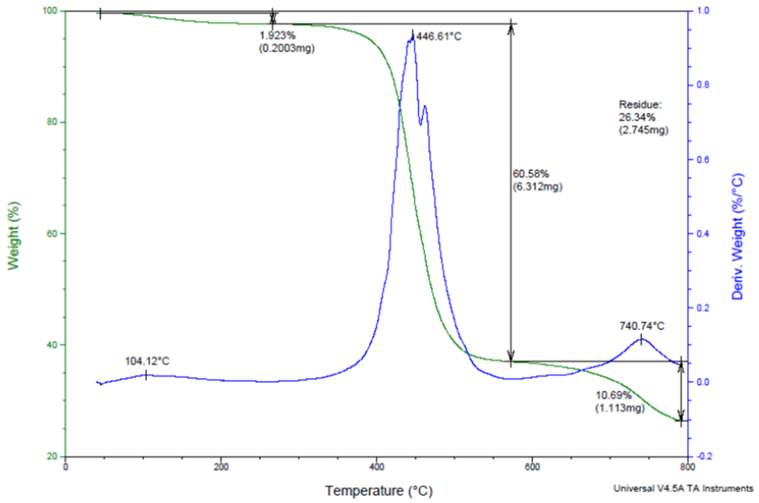

Figure S11. TGA results of the dried raw NAS.

5. Mass spectrometry (MS)

MS was performed on the three samples to analyze whether the samples contain substances with a low molecular weight. MS analyses were performed on an HP 5890 Series II gas chromatograph (Hewlett-Packard, Les Ulis, France).

The MS results of AMS sample are shown in Figure S12, mainly exhibiting the ion peaks of sodium petroleum sulfonate; specifically, the peaks at 397, 401, 403, 405, 407, and 409 belong to phenanthrenes (anthracenes), acenaphthenes, alkyl naphthalenes, benzodiacyclohexanes, indans, and alkylbenzenes, respectively.

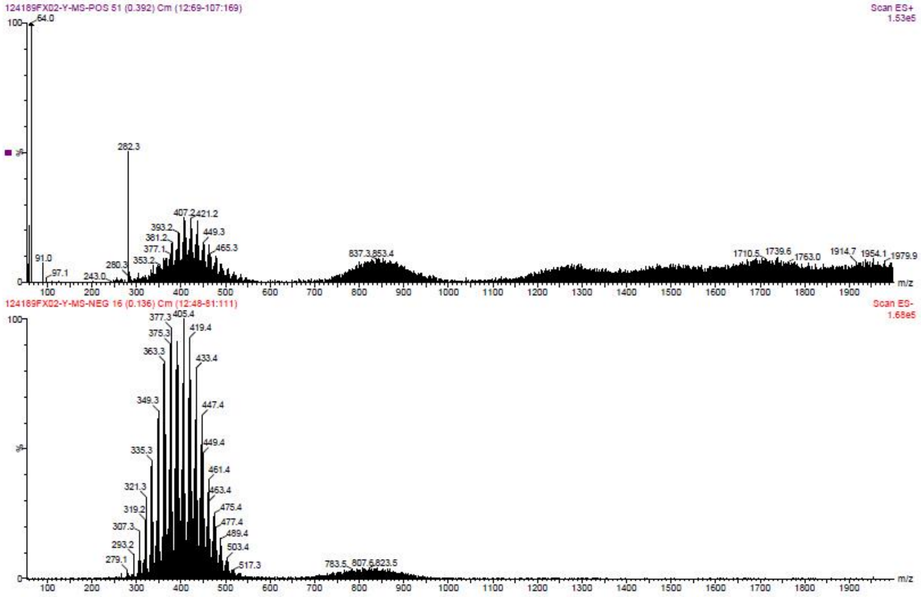

Figure S12. MS results of AMS.

The MS results of ADS are shown in Figure S13, which mainly present the ionic parameters of sodium petroleum sulfonate; specifically, the peaks at 367, 381, 395, 409, 423, 437, 451, and 465 belong to C_{15}-sodium alkyl benzene sulfonate, C_{16}-sodium alkyl benzene sulfonate, C_{17}-sodium alkyl benzene sulfonate, C_{18}-sodium alkyl benzene sulfonate, C_{19}-sodium alkyl benzene sulfonate, C_{20}-sodium alkyl

benzene sulfonate, C_{21}-sodium alkyl benzene sulfonate, and C_{22}-sodium alkyl benzene sulfonate. Meanwhile, peaks at 204, 218, and 246 are from acenaphthenes.

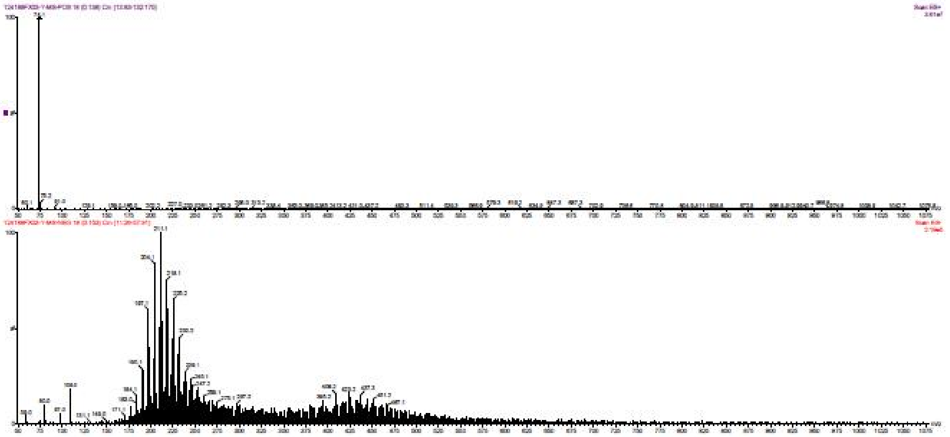

Figure S13. MS results of ADS.

The MS results of the raw NAS are shown in Figure S14, which mainly presents the ionic parameters of sodium petroleum sulfonate; specifically, the peaks at 397, 401, 403, 405, 407. and 409 belong to phenanthrenes (anthracenes), acenaphthenes, naphthalenes, benzodiacyclohexane, indans, and alkylbenzenes, respectively.

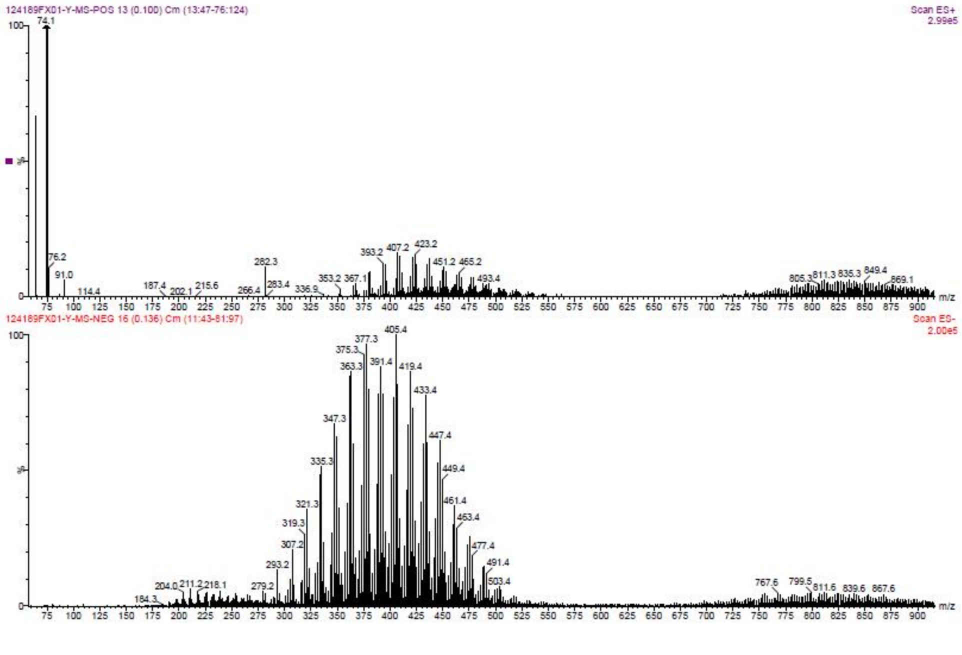

Figure S14. MS results of raw NAS.

6. Aggregation Number

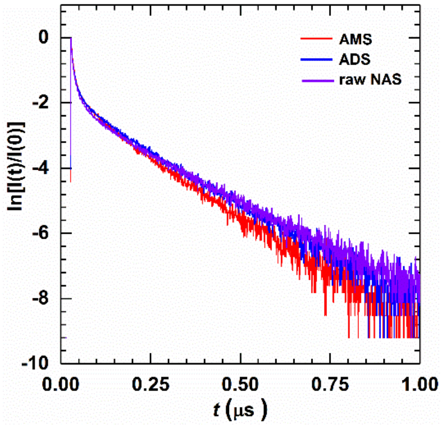

Figure S15. Decay profiles for pyrene fluorescence quenching in micelles.

7. Micellar or particle size

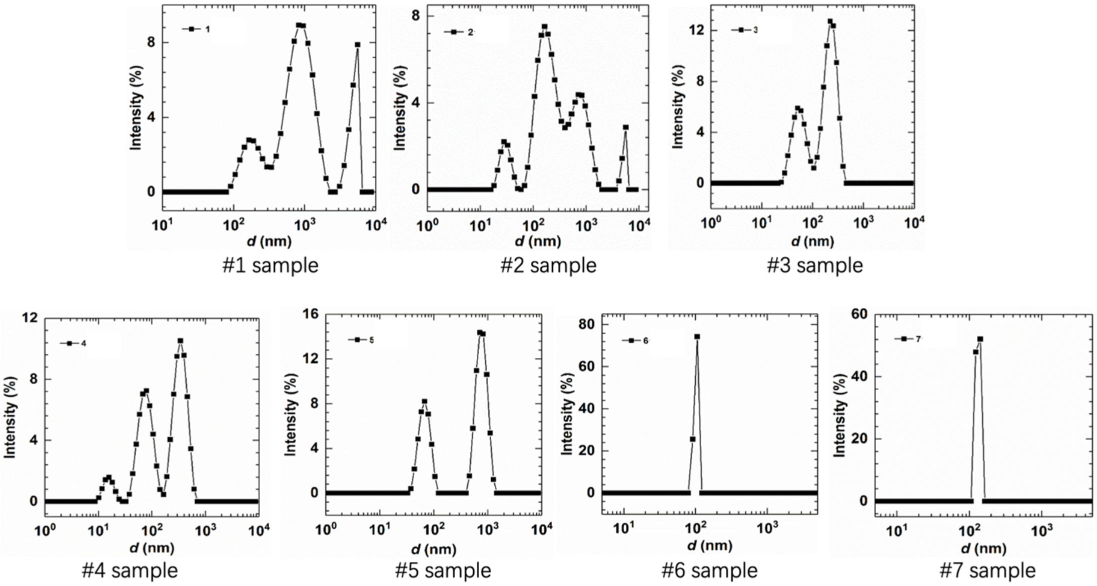

Figure S16. Effects of processing time on the micellar or particle size of samples #1–#7 collected from Karamay Oilfield.
